# The Weather

**Published:** 1870-01

**Authors:** 


### THE WEATHER.

Very many diseases are laid at the door of "the weather." It is the want of weather which brings multitudes in our larger cities to an untimely grave. "The weather" refers to the state of the out-door air; it may be cold or hot, wet or dry, serene or stormy; but it is all weather, provided it is out of doors. Our wits are stimulated in winter to keep "the weather" out of doors; and we eat, and sleep, and lounge in an atmosphere of sixty, seventy and eighty degrees, and that atmosphere loaded with impurities of human exhalations, tainted breaths, coal gas, furnace heat, kitchen effluvia. It is not wonderful that, breathing all these from morning until night, and until the morning comes again, with short intervals of an hour or two now and then, during all the weary months of winter, that our children pale and pine away, and by the coming of the beautiful spring-time, have such little vitality, that croup, and scarlet fever, and putrid sore throats sweep them by multitudes into an untimely grave. The best health invigorator that can be taken, suitable for all classes and conditions, is two hours of "weather" daily, rain or shine.

